# Next wave of interventions to reduce under-five mortality in Rwanda: a cross-sectional analysis of demographic and health survey data

**DOI:** 10.1186/s12887-018-0997-y

**Published:** 2018-02-05

**Authors:** Cheryl L. Amoroso, Marie Paul Nisingizwe, Dominique Rouleau, Dana R. Thomson, Daniel M. Kagabo, Tatien Bucyana, Peter Drobac, Fidele Ngabo

**Affiliations:** 1 Inshuti Mu Buzima/Partners in Health-Rwanda, Rwinkwavu, Rwanda; 2 USAID Global Health Fellows II, Public Health Institute, Washington DC, USA; 3 School of Public Health, College of Medicine and Health Science, University of Rwanda, Kigali, Rwanda; 4 Department of Global Health and Social Medicine, Harvard Medical School, Boston, MA USA; 5 Rwanda Ministry of Health, Kigali, Rwanda; 6 Division of Global Health Equity, Brigham and Women’s Hospital, Boston, MA USA

**Keywords:** U5M, U5MR, Under-five mortality, DHS, Africa

## Abstract

**Background:**

Sustained investments in Rwanda’s health system have led to historic reductions in under five (U5) mortality. Although Rwanda achieved an estimated 68% decrease in the national under U5 mortality rate between 2002 and 2012, according to the national census, 5.8% of children still do not reach their fifth birthday, requiring the next wave of child mortality prevention strategies.

**Methods:**

This is a cross-sectional study of 9002 births to 6328 women age 15–49 in the 2010 Rwanda Demographic and Health Survey. We tested bivariate associations between 29 covariates and U5 mortality, retaining covariates with an odds ratio *p* < 0.1 for model building. We used manual backward stepwise logistic regression to identify correlates of U5 mortality in all children U5, 0–11 months, and 12–59 months. Analyses were performed in Stata v12, adjusting for complex sample design.

**Results:**

Of 14 covariates associated with U5 mortality in bivariate analysis, the following remained associated with U5 mortality in multivariate analysis: household being among the poorest of the poor (OR = 1.98), child being a twin (OR = 2.40), mother having 3–4 births in the past 5 years (OR = 3.97) compared to 1–2 births, mother being HIV positive (OR = 2.27), and mother not using contraceptives (OR = 1.37) compared to using a modern method (*p* < 0.05 for all). Mother experiencing physical or sexual violence in the last 12 months was associated with U5 mortality in children ages 1–4 years (OR = 1.48, *p* < 0.05). U5 survival was associated with a preceding birth interval 25–50 months (OR = 0.67) compared to 9–24 months, and having a mosquito net (OR = 0.46) (*p* < 0.05 for both).

**Conclusions:**

In the past decade, Rwanda rolled out integrated management of childhood illness, near universal coverage of childhood vaccinations, a national community health worker program, and a universal health insurance scheme. Identifying factors that continue to be associated with childhood mortality supports determination of which interventions to strengthen to reduce it further. This study suggests that Rwanda’s next wave of U5 mortality reduction should target programs in improving neonatal outcomes, poverty reduction, family planning, HIV services, malaria prevention, and prevention of intimate partner violence.

## Background

Millennium Development Goal Four (MDG4) called for a two-thirds reduction in under five (U5) mortality between 1990 and 2015. Progress toward this goal was made worldwide, with the number of U5 deaths declining from nearly 12 million in 1990 to 6.9 million in 2011 [1]. However, improvement in Sub-Saharan Africa (39% reduction in mortality) was slower than most other regions including Northern Africa (68%) and Latin American and the Caribbean (64%), resulting in a widening disparity where 1 in 9 sub-Saharan African children still died before the age of five [1]. In contrast to regional trends, Rwanda achieved an estimated 70% decrease in the national U5 mortality rate between 2000 and 2011 [2]. Data suggest this could be the most rapid reduction of its kind ever documented, and as a result, Rwanda was one of a few low income countries to meet MDG4 by 2015 [1, 2].

With the establishment of the Millennium Development Goals (MDGs), the United Nations Millennium project published a list of immediately implementable “quick impact initiatives” that could result in major short-term gains in health for relatively low cost [3]. Like many countries in sub-Saharan Africa [4], Rwanda’s Health Sector Strategic Plan includes many such interventions, however these were integrated into a longer-term strategy, and included the elimination of user fees for some health services, the expansion of access to sexual and reproductive health information and services, and the training and support of community health workers [3, 5].

Following the devastating effects of civil war from 1990 and genocide in 1994, under-5 mortality was at its highest recorded in Rwanda, the economy was nearly destroyed, and the health system had collapsed. Rebuilding of the country and its systems began in 1999, and in 2000, Rwanda launched its ambitious “Vision 2020” plan [6], which laid out a 20-year road map for development, including pro-poor policies for growth to benefit the worst off. The education sector reform included in 2008 a special Girls Education Policy [7], aimed at “the progressive elimination of gender disparities in education and training,” focusing on access, quality and retention. The government had also prioritized gender parity in secondary and university education [8]. In the health sector, significant strategic investments were made to decentralize infrastructure and human resources in health, with the ratio of doctors and nurses to population achieved actually surpassing initial targets [5]. Explanations for Rwanda’s rapid reduction in U5 mortality have been detailed in the literature [2, 9], and center on development of a system with ready access and accountability, universal access to insurance, performance based financing, community health workers and coordinated use of foreign investment to strengthen health delivery systems.

While short- and long-term interventions appear to be having major impacts on U5 mortality in Rwanda, and while Rwanda was able to reach MDG4, it is an opportune moment to take stock and consider how to target future investments to maximize their impact. This article aims to identify areas for potential further intervention by evaluating socio-demographic and health factors associated with U5 mortality in the 2010 Rwanda Demographic and Health Survey (RDHS).

## Methods

### Data

This analysis is based on data collected from 6328 women age 15 to 49 in the 2010 RDHS and who had a child in the last five years (9002 births). The 2010 RDHS is a nationally representative two-stage cluster survey conducted roughly every five years. The survey was stratified by Rwanda’s 30 districts, with *imidugudu* (rural villages and urban neighborhoods) serving as primary sampling units, and oversampling in urban areas. The RDHS questionnaires [10] were translated into Kinyarwanda and back translated into English, and field tested before implementation. Data were collected between September 26, 2010 and March 10, 2011. The response rate for the 2010 DHS survey was 99% [10].

The primary outcome was mortality of children under age five. Complete birth histories were collected including month and year of each biological child’s birth and death. These data were used to identify the number of children born in the last five years, length of birth intervals, and child’s age at death. For each birth, the woman was additionally asked whether she wanted to be pregnant at that time, place of delivery, and approximate size of the baby at birth. For the most recent birth, women reported detailed information about antenatal care, including number and timing of antenatal visits.

**Fig. 1:**
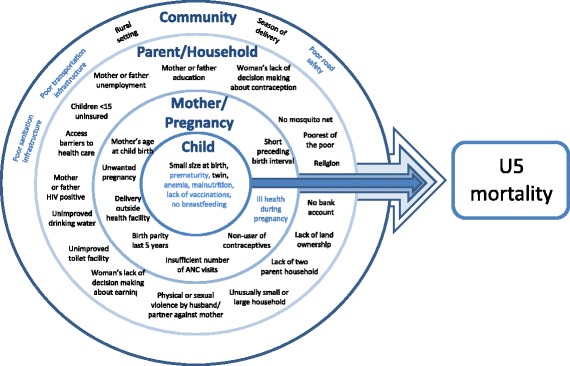
Conceptual framework of factors associated with U5 mortality

A literature search using PubMed, Google Scholar and HINARI was undertaken to identify biological and social determinants of neonatal, infant, and child mortality in Sub-Saharan Africa, and summarized in a conceptual framework (Fig. 1). All women reported demographic information including date of birth, marital status, religion, level of education and economic information including employment status, ownership of land, and dependency on others for economic decision-making. All women were asked about their current method of contraceptive use, individual health insurance coverage, as well as perceived barriers to care, including needing permission to go to the doctor, needing money for advice or treatment, distance to the health facility or not wanting to go alone. Women were asked antenatal care questions about their last pregnancy only. A random subset of one woman per household were invited to participate in a domestic violence survey in which they answered questions about physical, sexual, and emotional violence by a husband or partner in the last 12 months. A different randomized subset of women were measured for height and weight by the interviewer, and asked to provide a blood sample for HIV testing. Household size and configuration were calculated from a roster of household members. Multiple questions about household assets (e.g. access to treated drinking water, access to a bank account, and ownership of goods such as radio or bike) were used in a principle components analysis to generate a household wealth factor score [11], and those households in the left tail of the distribution (score < − 0.8) were classified as the poorest of the poor.

Per the survey design, father social and economic data were only available in a fraction of households, and since father and mother indicators (e.g. education) were highly correlated, father data were not included. Information about underweight, stunting, diarrhea, respiratory infection, fever, and immunization history could not be included in this analysis because data were only collected about children who survived to the date of interview. Breastfeeding was not included because nearly all children (93.5%) were breastfed within one day of birth [5], and those children not reported as breastfeeding in the first day of life were overwhelmingly the same children who died in the first days of life, so they may not have survived long enough to be breastfed.

### Statistical analysis

Based on the conceptual framework, we defined 29 covariates and tested bivariate associations with U5 mortality, retaining those covariates with an odds ratio *p*-value< 0.1 for model building; spurious associations between unmeasured covariates and the outcome were ignored. We tested for collinear covariates (Pearson’s *r* > 0.6), though none were found. We ordered the 17 remaining covariates from most-to-least import based on the conceptual framework and used manual backward stepwise logistic regression to arrive at a reduced model. Additional models were fitted for infants (age 0 to 11 months) and children (age 12 to 59 months) because pregnancy factors were expected to be more strongly associated with younger deaths than older deaths. We did not model risk factors for neonatal mortality alone because too few pregnancy and delivery variables were available and too few observations were available to make the analysis meaningful. All models controlled for living in the poorest households, mother’s education, mother’s age at child’s birth, and marital status. The analysis was performed in Stata version 12 using survey commands to account for the complex sample design and to apply sampling weights.

## Results

Of the 9002 children born in the last five years, 518 (5.8%) had died; 46% of deaths occurred in the neonatal period (0–30 days), 35% in the post-neonatal or infant period (1 to 11 months), and 19% of deaths occurred in children age 1 to 5 years. In bivariate analysis (Table 1), the following 14 factors were associated with U5 mortality: small size at birth, mother having less than four antenatal care visits, preceding birth interval of more than 24 months, having 3 to 4 births in the last five years, twin birth, desired pregnancy, birth outside of a health facility, mother having no education, widowed mother, HIV positive mother, mother not current user of contraceptive method, physical or sexual violence by husband or partner in the last 12 months, not having a mosquito net, and having an unimproved source of drinking water (*p* < 0.1 for all).

**Table 1:** Bivariate associations between sociodemographic and health characteristics, and childhood mortality, Rwanda 2010

Socio-demographic factor	Weighted % (death rate)	No. of deaths in last five years	No. of children ever alive in the last five years	Odds Ratio	*p*-value
Child variables					
Child size at birth					
Normal or large size	5.4	403	7558	1.00	
Small size	7.1	100	1392	1.32	0.0157
Child is twin					
No	5.4	462	8734	1.00	
Yes	20.2	56	268	4.45	< 0.0001
Mother and pregnancy variables					
Number of antenatal visits during pregnancy					
4+ antenatal visits, well timed	2.4	36	1577	1.00	
4+ antenatal visits, poorly timed	3.3	22	662	1.40	0.2440
0–3 antenatal visits	3.5	140	4075	1.51	0.0426
Not asked	12.0	320	2674	5.63	< 0.0001
Preceding birth interval					
9–24 months	8.6	133	1592	1.00	
25–50 months	4.8	193	4036	0.54	< 0.0001
51+ months	4.7	50	1090	0.53	< 0.0001
First born, or only child	6.0	135	2253	0.68	< 0.0001
Number of births in last five years					
1–2 births	4.5	362	8150	1.00	
3–4 births	18.2	156	852	4.74	< 0.0001
Unwanted pregnancy					
Wanted	6.2	349	5669	1.00	
Unwanted	5.1	162	3318	0.81	0.0554
Place of delivery					
Health facility	5.1	319	6207	1.00	
Not health facility	7.0	190	2779	1.40	0.0021
Mother’s age at child’s birth					
< 21 years	6.8	60	864	1.00	
21–30 years	5.5	261	4837	0.80	0.1338
> 30 years	6.0	197	3301	0.87	0.3688
Parent and household variables					
Mother’s education					
No education	7.2	124	1702	1.00	
Educated	5.5	394	7300	0.75	0.0112
Mother’s marital status					
Never in union	4.5	24	536	1.00	
Living with partner	5.8	443	7690	1.32	0.2785
Widowed	8.6	18	220	2.00	0.0630
Not living with partner	6.0	33	556	1.37	0.3500
Women’s religion					
Religious, Christian	5.9	503	8687	1.00	
Religious, not-Christian or not Religious	5.3	15	305	0.90	0.7148
Mother’s HIV status					
HIV negative	6.3	268	4358	1.00	
HIV positive	13.8	23	170	2.38	0.0003
Not tested	5.1	227	4474	0.79	0.0174
Mother’s BMI					
Underweight (< 18.5)	9.3	19	205	0.66	
Normal weight (> = 18.5 to < 25)	6.3	213	3464	0.76	0.1190
Overweight / obese (> = 25)	7.2	59	835	0.52	0.3515
Not measured	5.0	227	4498	0.66	0.0136
Mother’s current contraceptive method					
Modern method	4.6	178	3988	1.00	
Traditional method	5.9	33	528	1.29	0.2602
Non-User	6.9	307	4486	1.52	< 0.0001
Mother’s perceived barrier to care (permission, money, distance and not going alone)					
No	5.5	178	3245	1.00	
Yes	6.0	340	5757	1.10	0.3500
Physical or sexual violence by husband against mother, in last 12 months					
No	5.7	86	1559	1.00	
Yes	7.4	156	2171	1.33	0.5132
Not interviewed	5.2	275	5264	0.92	0.5132
Decision maker for using contraceptives					
Woman	6.1	19	340	1.00	
Husband	2.9	5	146	0.47	0.1443
Joint decision	4.7	170	3694	0.75	0.2890
Decision maker for woman’s earnings					
Woman and husband	5.8	190	3280	1.00	
Woman alone	5.5	42	820	0.94	0.7198
Husband alone	5.2	41	775	0.87	0.4494
Mother’s employment					
Not working	6.5	119	1853	1.00	
Working	5.6	398	7141	0.85	0.2004
Mother owns agriculture land					
Does not own land	5.2	115	2313	1.00	
Owns land	6.0	403	6686	1.17	0.1872
Poorest of the poor (wealth factor score < −0.8)					
No	5.8	506	8869	1.00	
Yes	9.3	12	133	1.68	0.1331
Household has a mosquito bed net for sleeping					
No	10.8	63	587	1.00	
Yes	5.5	455	8415	0.48	< 0.0001
Source of drinking water					
Unimproved	6.5	161	2480	1.00	
Improved	5.5	348	6431	0.83	0.0848
Household toilet facility					
Unimproved	6.2	144	2342	1.00	
Improved	5.6	365	6560	0.91	0.3923
Children < 15 have health insurance in household					
No	5.7	116	2098	1.00	
Yes	5.8	402	6904	1.02	0.8750
Household has bank account					
Yes	5.5	155	2914	1.00	
No	6.0	361	6062	1.09	0.4139
Household size					
2–3 members	5.6	203	1272	0.76	0.1335
4–5 members	4.3	53	3697	1.00	
6+ members	6.5	262	4025	1.17	0.1180
Community variables					
Season of delivery					
Short dry	5.9	120	2037	1.00	
Long rain	5.9	145	2484	1.00	0.9878
Long dry	6.3	147	2349	1.07	0.5915
Short rain	5.1	106	2132	0.85	0.2312
Place of residence					
Urban	5.6	64	1225	1.00	
Rural	5.9	454	7777	1.05	0.7137

In the reduced model of all children under five, the following factors were positively associated (predictive direction) with U5 mortality: household being among the poorest of the poor (OR = 1.98, *p* < 0.05), child being a twin (OR = 2.40, *p* < 0.001), mother having 3 or 4 births in the past 5 years (OR = 3.97, p < 0.001) compared to 1 or 2 births, mother being HIV positive (OR = 2.27, *p* < 0.01), and mother not using contraceptives (OR = 1.37, *p* < 0.01) compared to using a modern method. Analysis of childhood mortality by age group (Table 2) indicated that all factors associated with mortality in the combined-ages model were also associated for children age 1 to 4, but only mother having 3 or 4 births in the last five years was positively associated with mortality in infants aged 0 to 11 months. Different factors were negatively associated with mortality (e.g. associated with survival of children under five): preceding birth interval between 25 and 50 months (OR = 0.67, *p* < 0.01) compared to 9 to 24 months, and household having a mosquito net (OR = 0.46, *p* < 0.001); the factors were only associated with survival in children age 1 to 4, and not infants. Additionally, mother experiencing physical or sexual violence in the last 12 months was associated with mortality in children age 1 to 4 (OR = 1.48, *p* < 0.05).

**Table 2:** Multivariable odds ratios of childhood mortality by age group

Potential predictors	Under Five		Infant (0–11 months)		Child (12–59 months)	
	Full	Reduced	Full	Reduced	Full	Reduced
Poorest of the poor						
No	1.00	1.00	1.00	1.00	1.00	1.00
Yes	2.17*	1.98*	3.40	3.28	2.05*	1.82
Mother’s education						
No education	1.00	1.00	1.00	1.00	1.00	1.00
Educated	0.88	0.84	1.26	1.25	0.87	0.83
Mother’s age at child’s birth						
< 21 years	1.00	1.00	1.00	1.00	1.00	1.00
21–30 years	0.95	0.85	0.98	0.93	0.96	0.90
> 30 years	1.23	0.99	0.93	0.85	1.27	1.12
Mother’s marital status						
Single	1.00	1.00	1.00	1.00	1.00	1.00
Living with partner	0.89	1.49	0.59	0.68	0.95	1.46
Widowed	1.26	1.96	1.23	1.57	1.29	1.83
Not living with partner	1.02	1.43	0.29	0.27	1.11	1.39
Child size at birth						
Normal or large size	1.00		1.00		1.00	
Small size	1.15		1.78		1.12	
Child is twin						
No	1.00	1.00	1.00		1.00	1.00
Yes	1.85*	2.40***	2.00		1.97*	2.28***
Number of antenatal visits during pregnancy						
4+ antenatal visits, well timed	1.00		1.00		1.00	
4+ antenatal visits, poorly timed	1.13		0.54		1.31	
0–3 antenatal visits	1.29		0.86		1.50	
Not asked	4.01***		2.76		3.88***	
Preceding birth interval						
9–24 months	1.00	1.00	1.00		1.00	1.00
25–50 months	0.67**	0.67**	0.50		0.7*	0.7*
51+ months	0.8	0.70	0.75		0.80	0.69
First born, or only child	0.94	1.03	0.37		1.04	1.11
Number of births in last five years						
1–2 births	1.00	1.00	1.00		1.00	1.00
3–4 births	2.83***	3.97***	2.43	3.03*	3.12***	4.62***
Unwanted pregnancy						
Wanted	1.00		1.00		1.00	
Unwanted	1.02		0.64		1.10	
Place of delivery						
Health facility	1.00		1.00		1.00	
Not health facility	1.17		1.39		1.12	
Mother’s HIV status						
HIV negative	1.00	1.00	1.00		1.00	1.00
HIV positive	2.41**	2.27**	1.26		2.53***	2.29**
Not tested	0.79	0.77*	0.49		0.85	0.77
Mother’s current contraceptive method						
Modern method	1.00	1.00	1.00		1.00	1.00
Traditional method	1.33	1.31	0.96		1.36	1.36
Non-User	1.28*	1.37**	2.13		1.29*	1.47***
Physical or sexual violence by husband against mother in last 12 months						
No	1.00		1.00		1.00	1.00
Yes	1.3		0.61		1.42*	1.48*
Not interviewed	1.16		1.10		1.15	1.29
Mother owns agriculture land						
Does not own land	1.00		1.00		1.00	
Owns land	1.26		1.68		1.21	
Household has a mosquito bed net for sleeping						
No	1.00	1.00	1.00		1.00	1.00
Yes	0.41***	0.46***	0.23**	0.27**	0.44***	0.46***
Source of drinking water						
Unimproved	1.00					
Improved	0.93		1.18		0.91	
Household size						
2–3 members	1.09		1.36		1.03	
4–6 members	1.00		1.00		1.00	
7+ members	0.96		0.57		1.02	

Key: *p < 0.05; **p < 0.01; ***p < 0.001

## Discussion

Despite major reductions in under-five mortality in Rwanda, the percentage of children that do not survive to their first birthday remains too high, with the highest risk of mortality in the neonatal period. This analysis identified several factors associated with mortality in children under age five in Rwanda, and these findings point toward ways to build on existing interventions to reduce risk of mortality, particularly for infants.

### Poverty reduction

The fight against poverty in Rwanda has been impressive, with over a million people lifted out of poverty in the five-year period between 2006 and 2011; during this period income inequality, as measured by the Gini coefficient, declined [12]. Programs such as the national community-based health insurance, which includes fee exceptions for the poorest of the poor, have helped remove financial barriers to care for the most vulnerable. Analysis of the progress in child survival over the past two decades has found that it occurred with increasing social equity, including a reduction in differences among household wealth groups, education levels and between rural and urban areas) [9, 13]. Despite improvements, these results showed that Rwanda’s poorest families are still nearly twice as likely to experience the death of a child under five. Still more needs to be done to reduce risks for mortality among the poor and marginalized.

### Access to family planning

Effective investments to reduce U5 mortality in Rwanda should support contraceptive use and encourage healthy birth spacing. This study found that more numerous and closely spaced births are a risk factor for U5 mortality, which is similar to results from multi-country studies [14, 15]. Overall fertility rates declined sharply in Rwanda between 2005 and 2010 falling from 6.1 to 4.6 births per woman [16, 17]., and then to 4.2 in 2015. This has been attributed to a national political shift in Rwanda toward promotion of smaller families [18] and a dramatic expansion in contraceptive usage from 10.3% in 2005 to 45.1% in 2010 [10]; one of the fastest increases in modern method uptake ever reported [9, 18]. Despite these trends, 19% of married women in Rwanda reported an unmet need for family planning [18]. Close birth spacing and unintended pregnancies can contribute to U5 mortality in several ways, including the harmful effects of the early child weaning, “maternal depletion syndrome,” which weakens mothers and can result in low birth weight and prematurity, and the drain on household resources, including food, that comes with an additional member [19]. A study in Kenya estimated that mortality would decline 11% for neonates, 13% for infants and 17% for all children under-five simply by meeting all of the contraceptive needs of women [20]. These results suggest that targeting the unmet contraceptive need of Rwandan women could reduce risk of U5 and maternal mortality. Continued increases in access and use of contraception are a part of the country’s strategic plan for health improvement and a clearly stated priority of the Ministry of Health [5, 17].

### HIV services

HIV rates in Rwanda are low compared to other sub-Saharan African countries; the adult prevalence in Rwanda is 3%, compared to neighboring countries like Tanzania (5%) and Uganda (7%) [21]. Children of seropositive mothers are at risk of HIV infection during the pregnancy, delivery and breastfeeding [22]. Early infant testing and diagnosis is of vital importance and requires close post-natal follow-up, as over half of HIV positive children without treatment die, most within their first six months of life [22]. Children of seropositive mothers face additional risks, such as the greater likelihood of being born with low birth weight, exposure to contaminated drinking water during formula feeding, and the potential social and economic isolation faced by their mothers [23]. Our results showed that the mothers of 3.9% of children in Rwanda tested positive for HIV, and these mothers had more than twice the odds of losing a child under age five than HIV negative mothers. The decentralization of the Rwandan healthcare system and the development of a maternal health focused community health program have both helped encourage mothers to complete antenatal care (ANC) visits, which are crucial for the early detection and initiation of treatment. However, despite consistently high levels of seeking ANC (94% in 2005 and 98% in 2010), expectant mothers still rarely (35%) complete the minimum number set by World Health Organization standards and tend to initiate them late (62%) [10]. Nevertheless, rates of facility births have improved impressively, from 28% in 2005 to 69% in 2010 [10]. The Rwandan Ministry of Health also introduced the national B+ treatment program in 2011, with the aim of reducing mother-to-child HIV transmission through the commencement of lifelong antiretroviral triple therapy during pregnancy regardless of clinical stage, coupled with exclusive breastfeeding. These trends and new programs are promising, yet more needs to be done for early detection, close follow-up, as well as to mitigate the other risks children face with seropositive mothers.

### Malaria prevention

Tremendous strides have been made in malaria control and treatment globally with the cumulative probability of death due to malaria falling from 35.8 to 12.3 per 1000 children under five between 1980 and 2010 [24]. Malaria is estimated to cause 18% of deaths among children under five in sub-Saharan Africa [25], and is an important cause of U5 mortality in Rwanda [26]. A meta-analyses by Eisele and colleagues found a protective effect of insecticide treated nets (ITNs) for reducing all-cause mortality by 18% among children aged 1 to 59 months [27]. In this current study, children living in a household with a mosquito bed net had half the odds of mortality compared to those who do not, suggesting that a strong national malaria control program with bed net distribution are important. In 2009, the government of Rwanda introduced community-level testing and treatment of malaria through the national Community Health Worker program. It is expected that the inclusion of malaria in a package of Integrated Management of Childhood Illnesses (IMCI), in addition to regular distribution of treated mosquito nets, will lead to decreased mortality for children under 5. Rwanda has recently experienced an increase in malaria cases, reportedly due to a substantial decline in the use of ITNs [28]. Renewed efforts toward effective malaria prevention, particularly ITNs, will be critical for Rwandan’s U5 mortality prevention efforts.

### Empowering women by addressing intimate partner violence

A link between intimate partner violence (IPV) and mortality in children has been found in several low income countries, including Rwanda, though this link is not universal [24]. Possible hypotheses are that violence against women is related to violence against children, or that violence is part of a larger disempowerment of women that would limit access to resources and services [29]. The latter theory is reinforced by preliminary qualitative analyses of U5 death verbal autopsies conducted in rural Rwanda (unpublished data from the Verbal and Social Autopsy Study). The portion of women reporting ever experiencing physical or sexual IPV in Rwanda increased sharply from 34% in 2005 to 56% in 2010 [9, 16]. This increase in reported IPV could reflect an actual increase in violence, possibly linked with disruption of traditional gender roles associated with improvements in women’s education, employment, and political representation that have been achieved in Rwanda in the last decade [30, 31]. Alternatively, the increase might reflect improved reporting due to increased women’s empowerment [30], or recent legal and institutional changes around gender-based violence (GBV), including a new 2008 law on the prevention and punishment of GBV (No. 59/2008) and the creation of gender-desks in police stations staffed mostly by women [32]. The Rwandan government has implemented a variety of gender based violence prevention programs including prevention clubs in schools and universities, and Gender Based Violence committees at the village level, which aim to improve people’s knowledge about their rights and support reporting of violence. The Isange One Stop Center program, which offers integrated medical care, psychosocial support, and legal support for victims of domestic violence is currently being scaled to all district hospitals nationwide by 2018. Addressing IPV and its health impacts is challenging, therefore these centers could prove to be an important part of the solution.

Finally, the high proportion of under-five deaths occurring in the neonatal period (46%) suggests the need for particular focus on interventions to improve neonatal survival. Although the percentage of Rwandan women delivering in a health facility increased from 28% in 2005 to 69% in 2010 [10], evidence suggests numerous gaps in the quality of facility-based care during delivery and the early neonatal period [33]. Evidence-based policies and programs to improve neonatal care are underway, and this is an important area for future study [34].

Limitations of this study include the inability to examine factors that were not included in the Rwanda DHS, as well as the retrospective nature of the death reporting which could lead to recall bias and prevents comparison of individual characteristics such as anemia or stunting. In particular, nutrition likely plays a major contributing role in mortality but could not be examined with the existing data. In addition, though important, father’s data could not be included because men were only interviewed in one of every three households, resulting in large amounts of missing data among fathers. Because women were only asked antenatal care questions about the last birth, the higher rate of mortality in those births that were “not asked” about suggests interviewer or reporter bias to avoid talking about the child who died, and/or underreporting or misreporting the timing of recent deaths. The inability to separately examine factors associated with neonatal mortality may miss critical improvements needed for decreasing neonatal deaths. While the available DHS data did not allow this analysis, the authors are currently completing research on specific contexts and factors associated with neonatal death through verbal autopsy, which is expected to provide additional information to target neonatal death reduction specifically, in the Rwandan context. Additional confounding factors may be present that were not controlled for through the selected RDHS data. Finally, certain social characteristics such as the household wealth and urban/rural residence reflect the family situation at the time of the survey, and may have been different at the time of the child’s death [9].

## Conclusion

A number of programs have led to massive improvements in under-five mortality in Rwanda, including IMCI, near universal coverage of childhood vaccinations, a national community health worker program, and a near-universal health insurance scheme. As the reductions in U5 mortality that can be achieved by these programs are realized, it is time to think about where to focus efforts and programs to further reduce childhood mortality. In addition to continuing and improving work specifically targeting neonatal mortality reduction, where gains have not been made as rapidly as for older ages, these results suggest that continued investment in family planning, HIV services, malaria prevention, and prevention and prosecution of IPV are key toward further reductions in child mortality. Careful study and comparison of the social determinants of U5 mortality with data from the next DHS survey is recommended to track this progress.

## Abbreviations

ANC: Antenatal care
GBV: Gender-based violence
IMCI: Integrated Management of Childhood Illnesses
IPV: Intimate partner violence
ITN: Insecticide-treated mosquito bed net
MDG: Millennium Development Goal
MDG4: Millennium Development Goal Four
RDHS: Rwanda Demographic and Health Survey
U5: Under five

## References

[1] United Nations Children's Fund (UNICEF). Levels & trends in child mortality: report 2012. New York (NY): UNICEF; 2012. Available from: www.unicef.org/videoaudio/PDFs/UNICEF_2012_child_mortality_for_web_0904.pdf.

[2] Farmer PE, Nutt CT, Wagner CM, Sekabaraga C, Nuthulaganti T, Weigel JL (2013). Reduced premature mortality in Rwanda: lessons from success. BMJ.

[3] UN Millennium Project. Investing in development: a practical plan to achieve the millennium development goals. New York: United Nations Development Programme; 2005. Available from: http://www.who.int/hdp/publications/4b.pdf.

[4] World Bank, UNICEF, UNFPA, Partnership for Maternal, newborn and child health (2009). Health systems for the millennium development goals: country needs and funding gaps.

[5] [Rwanda] Ministry of health (MOH). Third health sector strategic plan July 2012 – June 2018. Kigali: MOH; 2012. Available from: www.moh.gov.rw/fileadmin/templates/Docs/HSSP_III_FINAL_VERSION.pdf.

[6] [Rwanda] Ministry of Finance and Economic Planning. Rwanda vision 2020. Kigali: Government of Rwanda; 2000. Available from: www.minecofin.gov.rw/fileadmin/templates/documents/NDPR/Vision_2020_.pdf.

[7] [Rwanda] Ministry of Education (2008). Girls' education policy.

[8] Government of Rwanda (2009). Report on the implantation of the Beijing declaration and platform for action (1995) and the outcome for the twenty-third special session of the general assembly (2000).

[9] Musafili A, Essén B, Baribwira C, Binagwaho A, Persson LA, Selling KE (2015). Trends and social differentials in child mortality in Rwanda 1990–2010: results from three demographic and health surveys. J Epidemiol Community Health.

[10] National Institute of Statistics of Rwanda (NISR), [Rwanda] Ministry of Health (MOH), and ICF International. Rwanda Demographic and Health Survey 2010. Calverton (MD): NISR, MOH, and ICF International; 2012. Available from: http://dhsprogram.com/pubs/pdf/fr259/fr259.pdf.

[11] Rutstein SO. The DHS Wealth index: approaches for rural and urban areas. DHS working paper no. 60. Calverton: Macro International, Inc.; 2008. Available from: http://dhsprogram.com/pubs/pdf/WP60/WP60.pdf.

[12] National Institute of Statistics of Rwanda (NISR). The third integrated household living conditions survey. Kigali: NISR; 2012. Available from: http://www.statistics.gov.rw/publication/eicv-3-main-indicators-report.

[13] McKinnon B, Harper S, Kaufman JS, Bergevin Y (2014). Socioeconomic inequality in neonatal mortality in countries of low and middle income: a multicountry analysis. Lancet Glob Health.

[14] Rutstein SO (2005). Effects of preceding birth intervals on neonatal, infant and under-five years mortality and nutritional status in developing countries: evidence from the demographic and health surveys. Int J Gynaecol Obstet.

[15] Rutstein SO (2008). Further evidence of the effects of preceding birth intervals on neonatal, infant, and under-five-years mortality and nutritional status in developing countries: evidence from the demographic and health survey. Working paper no. 41.

[16] Institut National de la Statistique du Rwanda (INSR), ORC Macro Rwanda Demographic and Health Survey 2005. Calverton (MD): INSR, ORC Macro; 2006. Available from: http://dhsprogram.com/pubs/pdf/FR183/FR183.pdf.

[17] Westoff CF (2013). The recent fertility transition in Rwanda. Popul Dev Rev.

[18] Ministry of Finance and Economic Planning (MINECOFIN). Economic development and poverty reduction strategy 2013-2018. Kigali: Government of Rwanda; 2013. Available from: http://www.minecofin.gov.rw/index.php?id=149.

[19] Boerma JT, Bicego GT (1993). Preceding birth intervals and child survival: searching for pathways of influence. Stud Family Plann.

[20] Rafalimanana H, Westoff CF. Gap between preferred and actual birth intervals in sub-Saharan Africa: implications for fertility and child health. DHS analytical studies no. 2. Calverton (MD): ORC Macro; 2001. Available from: www.dhsprogram.com/pubs/pdf/AS2/AS2.pdf.

[21] UNAIDS (2013). Global report: UNAIDS report on the global AIDS epidemic 2013.

[22] Newell ML, Coovadia H, Cortina-Borja M, Rollins N, Gaillard P, Dabis F (2004). Mortality of infected and uninfected infants born to HIV-infected mothers in Africa: a pooled analysis. Lancet.

[23] Leroy V, Ladner J, Nyiraziraje M, De Clercq A, Bazubagira A, Van de Perre P (1998). Effect of HIV-1 infection on pregnancy outcome in women in Kigali, Rwanda, 1992–1994. AIDS.

[24] Rico E, Fenn B, Abramsky T, Watts C (2011). Associations between maternal experiences of intimate partner violence and child nutrition and mortality: findings from demographic and health surveys in Egypt, Honduras, Kenya, Malawi and Rwanda. J Epidemiol Community Health.

[25] Rowe AK, Rowe SY, Snow RW, Korenromp EL, JRMA S, Stein C (2006). Estimates of the burden of mortality directly attributable to malaria for children under 5 years of age in Africa for the year 2000 - final report.

[26] Liu L, Johnson HL, Cousens S, Perin J, Scott S, Lawn JE (2012). Global, regional, and national causes of child mortality: an updated systematic analysis for 2010 with time trends since 2000. Lancet.

[27] Eisele TP, Larsen D, Steketee RW (2010). Protective efficacy of interventions for preventing malaria mortality in children in plasmodium falciparum endemic areas. Int J Epidemiol.

[28] World Health Organization (WHO). World malaria report 2011. Geneva: WHO. p. 2011. Available from: http://www.who.int/malaria/world_malaria_report_2011/en/.

[29] Roman NV, Frantz JM (2013). The prevalence of intimate partner violence in the family: a systematic review of the implications for adolescents in Africa. Fam Pract.

[30] Burnet JE (2011). Women have found respect: gender quotas, symbolic representation, and female empowerment in Rwanda. Polit Gender.

[31] Rocca CH, Rathod S, Falle T, Pande RP, Krishnan S (2009). Challenging assumptions about womens empowerment: social and economic resources and domestic violence among young married women in urban South India. Int J Epidemiol.

[32] Thomson DR, Bah AB, Rubanzana W, Mutesa L (2015). Correlates of intimate partner violence against women during a time of rapid social transition in Rwanda: analysis of the 2005 and 2010 demographic and health surveys. BMC Womens Health.

[33] Ngabo F, Zoungrana J, Faye O, Rawlins B, Rosen H, Levine R (2012). Quality of Care for Prevention and Management of common maternal and newborn complications: findings from a National Health Facility Survey in Rwanda.

[34] Hansen A, Magge H, Labrecque M, Munyaneza RBM, Nahimana E, Nyishime M (2015). The development and implementation of a newborn medicine program in a resource-limited setting. Public Health Action.

